# MicroRNAs: key players of taxane resistance and their therapeutic potential in human cancers

**DOI:** 10.1111/jcmm.12131

**Published:** 2013-09-23

**Authors:** Shi-Yun Cui, Rui Wang, Long-Bang Chen

**Affiliations:** a Department of Medical Oncology, School of Medicine, Jinling Hospital, Nanjing UniversityNanjing, Jiangsu, China

**Keywords:** microRNA, taxane, chemoresistance, apoptosis, cell cycle, epithelial–mesenchymal transition

## Abstract

The successful long-term use of taxane for cancer therapy is often prevented by the development of drug resistance in clinic. Thus, exploring the mechanisms involved is a first step towards rational strategies to overcome taxane resistance. Taxane resistance-related microRNA (miRNAs) are under investigation and miRNAs could induce the taxane resistance of tumour cells by regulating cell cycle distribution, survival and/or apoptosis pathways, drug transports, epithelial–mesenchymal transition and cancer stem cell. This article summarizes current research involving miRNAs as regulators of key target genes for tanxanxe chemoresistance and discusses the complex regulatory networks of miRNAs. Also, the authors will envisage future developments towards the potential use of targeting miRNAs as a novel strategy for improving response of tumour patients to taxane. miRNAs play critical roles in taxane chemoresistance and the miRNA-based therapies will be helpful for overcoming drug resistance and developing more effective personalized anti-cancer treatment strategies. Further research studies should be performed to promote therapeutic–clinical use of taxane resistance-related miRNAs in cancer patients, especially in those patients with taxane-resistant cancers.

- Introduction
  – miRNAs involved in taxane resistance
  – miRNAs involved in regulation of taxane targets
  – miRNAs involved in cell cycle control of taxanes
  – miRNAs involved in taxane-induced survival and/or apoptosis pathways
  – miRNAs involved in regulation of drug transports
  – miRNA involved in regulation of EMT and cancer stem cell
- Discussion
- Conclusion
- Expert commentary
- Five-year view

## Introduction

The taxanes (paclitaxel and docetaxel) are microtubule-stabilizing agents that function primarily by interfering with spindle microtubule dynamics and causing cell cycle arrest and apoptosis. Currently, taxanes have been used in clinics for the treatment of several kinds of tumours such as ovarian, breast, head and neck, lung, and prostate cancers [1]. However, drug resistance represents a major obstacle to improve the long-term effectiveness of taxanes in cancer chemotherapy. The mechanisms underlying the taxane resistance have not yet been fully elucidated.

As an integrated part of the multilayered regulatory networks of gene expression, miRNAs have raised great interest for their unique and powerful regulatory effects. They are short (20–23-nucleotide), endogenous, single-stranded RNA molecules, which negatively regulate the gene expression by base-pairing interactions between the ‘seed’ region (positions 2–8 from the 5′ end) of the miRNA and the partially complementary region (usually in the 3′ untranslated region) of the target miRNA. An estimated 60% of the miRNAs involved in distinct cellular processes have one or more predicted binding sites to interact with miRNAs. Although the repression effect of a single miRNA on specific miRNA is relatively modest compared with the transcriptional factor, its capacity to modulate tens to hundreds of target genes makes it have profound influence in normal and malignant cell homoeostasis. The aberrance of miRNAs can finally lead to disease and tumourgenesis [2].

**Fig. 1:**
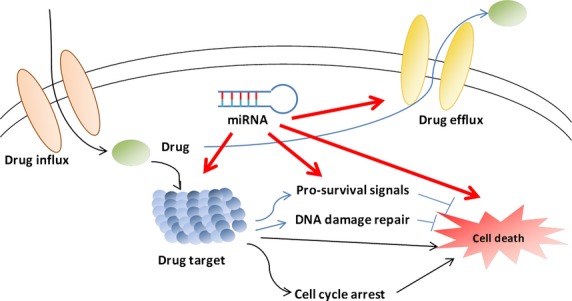
Roles of miRNAs in drug resistance. The mechanisms involved in drug resistance of cancer cell include alteration of drug target, altered regulation of the cell cycle and apoptosis, increased DNA damage repair and ejection of the drug from the cell by drug efflux pumps. miRNAs can influence the drug response by regulating all these cellular processes.

Abnormal expression of miRNAs has been observed in diverse haematological and solid tumours [3], as well as in chemoresistant cancer cells [4]. MicroRNAs can contribute to carcinogenesis through regulating multiple key cellular processes by functioning as oncogenes or tumour suppressors, depending upon the nature of their target gene (s). Those genes whose products play a role in the response to anti-cancer treatments are also likely to be regulated by miRNAs. As far as we know, there are a number of mechanisms involved in drug resistance of cancer cell, including alteration of drug target, altered regulation of the cell cycle and apoptosis, increased DNA damage repair and ejection of the drug from the cell by drug efflux pumps [5]. Indeed, recent evidence indicates that miRNAs can influence the effect of anti-cancer agents, including taxanes, through all the above mechanisms (Fig. 1), suggesting that they can play a pivotal role in drug efficacy and have potential clinical implications for overcoming taxane resistance. Therefore, this review aims to discuss the role of miRNAs in the molecular mechanisms of taxane resistance and the potential use of miRNAs in overcoming taxane resistance.

## MiRNAs involved in taxanes resistance

### MiRNAs involved in regulation of taxanes targets

The cellular target for paclitaxel has been identified as β-subunit of tubulin in microtubules. Docetaxel shares the same binding site as paclitaxel, although with greater affinity [6]. They both inhibit the process of cell division, causing a ‘frozen mitosis’ by stabilizing microtubules and thus preventing disassembly [7]. Mechanisms underlying taxane resistance that occur directly at the microtubule include mutations, alternations of tubulin isotype or regulatory proteins [8–10].

**Fig. 2:**
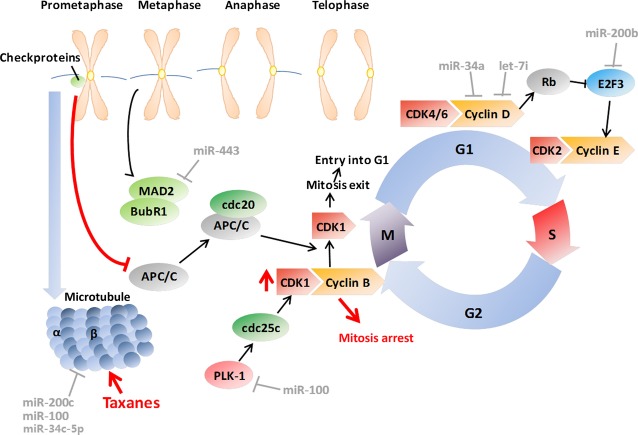
miRNAs involved in regulation of taxane targets and taxane-induced cell cycle change. Taxanes target β-tubulin in microtubules, recruit checkpoint proteins, such as MAD2, and suppress anaphase-promoting complex (APC/C), leading to increased activity of cyclin B1-CDK1 and cell cycle arrest at the M phase. Some key genes and their products in these pathways are under regulation of miRNAs, the aberrant expression of which could compromise the effect of taxanes.

Mutations in β-tubulin can disrupt microtubule assembly, conferring taxane resistance [11]. There are seven isotypes of β-tubulin in humans. β I and β IV-tubulin isotypes are constitutively expressed, while the other isotypes are tissue-specific [12]. Overexpression of β III-tubulin has been found to be associated with paclitaxel resistance in ovarian cancer [13], breast cancer [14], non small-cell lung cancer [15] and pancreatic cancer [16]. The possible mechanism was associated with reduced effects of paclitaxel on microtubule dynamic instability [17]. β III-tubulin is one of the direct targets of miR-200c, and restoration of miR-200c enhanced sensitivity to paclitaxel in endometrial, breast and ovarian cancer cell lines [18]. In addition to miR-200c, the other members of miR-200 family (miR-141, miR-200a, miR-200b, and miR-429) could down-regulate the expression of β III -tubulin in ovarian tumour, and might play a role as prognostic factors and markers of the response to paclitaxel-based chemotherapy in ovarian carcinoma [19]. On the other hand, β-tubulin miRNAs of classes I, IIA, IIB and V were proved to be regulated by miR-100 in MCF-7 breast cancer cells [20]. Microtubule-associated protein tau (MAPT) functions primarily by enabling tubulin assembly and microtubule stabilization [21]. Dysregulation of MAPT by miR-34c-5p was critical in the chemosensitivity of gastric cancer to paclitaxel [22] (Fig. 2).

### MiRNAs involved in cell cycle control of taxanes

Taxanes mainly induce mitotic arrest (G_2_/M arrest) and apoptosis by binding to the β–tubulin subunit of microtubules and suppressing dynamics of mitotic spindle [23]. The mitotic spindle assembly checkpoint (SAC) is activated when the attachment of the microtubules to the kinetochores is interrupted by taxanes [24]. Checkpoint proteins are then recruited, monitoring accurate microtubule attachment. The anaphase-promoting complex (APC/C) is suppressed by the mitotic checkpoint complex (MCC) formed by some checkpoint proteins, such as mitotic arrest deficiency protein 2 (MAD2), BUBR1 and Cdc20 until they leave the kinetochores when all microtubules attach to the kinetochores [25]. Activated by cdc20, the APC/C ubiquitin ligase ubiquitinates and degrades cyclin B1 and thereby inactivates cyclin-dependent kinase 1 (CDK1) in anaphase, allowing exit from mitosis to occur [26]. The sustained activation of checkpoint proteins by taxanes increases activity of cyclin B1-CDK1 through inhibiting APC/C, leading to cell cycle arrest at the M phase, which plays a key role in taxane function [27].

The MAD2 is a key component of the checkpoint proteins. Evidence shows that paclitaxel-induced activation of the SAC and apoptotic cell death requires MAD2 activity independent of MAD1 [28]. Reduced MAD2 expression was associated with increased resistance to paclitaxel in epithelial ovarian cancer (EOC) cells and poor outcome of high-grade serous EOC patients, and MAD2 was identified as a target of miR-433 [29]. Additionally, polo-like kinase 1 (PLK1) promotes mitotic progression by phosphorylating CDC25C, which in turn activates CDK1-Cyclin B1 complexes [30]. Inhibition of PLK1 by LFM-A13 could enhance the effects of paclitaxel [31]. Furthermore, PLK1-specific siRNAs and paclitaxel could exert synergistic effects in breast cancer by inducing inhibition of cell proliferation and increased apoptosis [32]. Overexpression of PLK1 has been found in a number of human tumours and PLK1 miRNA is proposed to be the target of miR-100 [33–35]. In our previous report, miR-100 has been shown to be a chemosensitivity restorer to docetaxel in human lung adenocarcinoma cells by targeting PLK1 [36].

Although taxanes mainly function in G_2_/M phase, aberrant changes occurring in G_1_/S phase also influence the taxane effects. Cyclin D1 binds to CDK4 and CDK6 to form a complex, supporting G_1_–S transition [37]. Kastl *et al*. reported that reduced cyclin D1 expression modulated by miR-34a could induce G1 phase arrest, where docetaxel exerts little cytotoxicity, in turn leading to the resistance of docetaxel in breast cancer cells [38]. Cyclin D1 is also a target of let-7i, and a chimera that combines Mucin 1 (MUC1) aptamer and let-7i miRNA was proved to reverse paclitaxel resistance in EOC cells through down-regulation of cyclin D1, cyclin D2, Dicer 1, and PGRMC1 expressions [39]. E2F family members play an important role in the control of gene expression in several phases of the cell cycle and in multiple checkpoints. Their targets are diverse and cover genes controlling DNA replication and G_1_/S transitions, such as Cyclin A/E, Cdc6, and Mcms, as well as products involved in DNA repair and G_2_/M transitions, such as Cdc25a, Cdk1, Aurora-A and Survivin [40]. In our previous study, E2F3 is also found to be a direct target of miR-200b, and inhibition of miR-200b, which led to E2F3 overexpression, contributed to resistance of lung adenocarcinoma cells to docetaxel [41] (Fig. 2).

Adenomatous polyposis coligene (APC) is a multi-functional tumour suppressor well known for its regulation of WNT signalling [42], and recent studies have identified its additional role of regulating cell adhesion and migration, microtubule networks, spindle formation and chromosome segregation [43]. APC deficiency could compromise the response to paclitaxel *in vivo* because it could lead to destabilized microtubules, independent of its effect on WNT signalling [44]. MiR-135a was associated with reduced APC in paclitaxel-resistant NSCLC cell lines and *in vivo* models, probably through interfering with the mitotic spindle checkpoint [45].

### MiRNAs involved in taxane-induced survival and/or apoptosis pathways

The cytotoxic effect on tumour cells of paclitaxel is demonstrated to depend on drug concentration, cell type and exposure time [46]. At low concentrations, paclitaxel induces mitotic arrest or an aberrant mitotic exit into a G_1_-like ‘multinucleate state’ ending up in apoptosis. Higher concentrations of paclitaxel can lead to extensive microtubule damage [7]. Independent of cell cycle arrest, paclitaxel induces apoptosis through multiple mechanisms, including activation of mitogen-activated protein kinases (MAPK) [47], Raf-1 [48, 49], and c-Jun N-terminal kinase (JNK) [50] and regulation of the expression of apoptosis-related proteins like Bcl-2, Bad, Bcl-xL, p21 WAF-1/CIP-1, and the tumour necrosis factor-related apoptosis-inducing ligand (TRAIL) receptors, DR4 and DR5 [48, 51, 52].

**Fig. 3:**
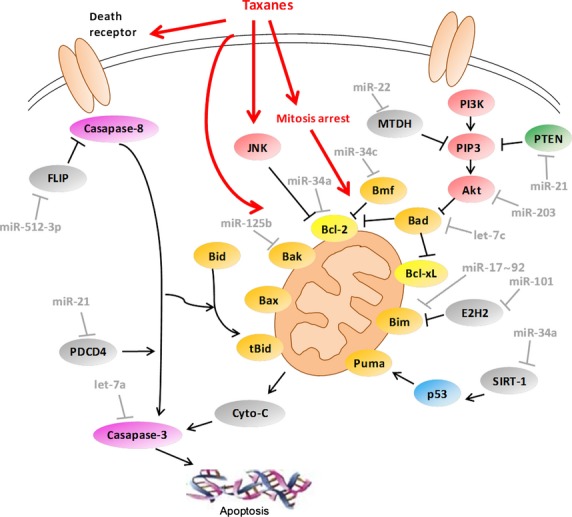
miRNAs involved in regulation of taxane-induced apoptosis. Taxanes induce cell apoptosis through both the intrinsic and extrinsic apoptosis pathways. Some key genes and their products in these pathways are under regulation of miRNAs, the aberrant expression of which could compromise the effect of taxanes.

A number of miRNAs have been reported to participate in the regulation of taxane-induced apoptosis (Fig. 3). MiR-512-3p facilitated paclitaxel-induced apoptosis mediated by death receptor (DR) through directly targeting cellular FLICE-like inhibitory protein (c-FLIP) in hepatocellular carcinoma cells [53]. By inhibiting casapase-8, c-FLIP plays an anti-apoptotic role in DR signalling [54]. Additionally, miR-34c-induced apoptosis is commonly found in several cancer cell lines [55]. However, as it might confer resistance to caspase-8- and paclitaxel-induced apoptosis in NSCLC cells, miR-34c also showed an oncogenic potential [56].

The Bcl-2 family plays a pivotal role in the mitochondrial apoptosis pathway and is considered associated with taxane-induced apoptosis. The development of drug resistance in various cancer cells has also been linked to abnormalities in the expression of Bcl-2 family proteins. The Bcl-2 family consists of two sets of proteins exerting opposite functions. The pro-apoptotic subfamily includes Bax, Bak and the BH3-only proteins includes Bid, Bim, Bad and PUMA, while the anti-apoptotic subfamily includes Bcl-2, Bcl-xL and McL-1 [57]. Bcl-2 has been demonstrated to be a target of miR-34a [58]. Interestingly, down-regulation of miR-34a was observed in paclitaxel-resistant prostate cancer, and introduction of miR-34a precursor attenuated resistance to paclitaxel [59]. In contrast, increased miR-34a expression was found in MCF-7 docetaxel-resistant breast cancer cells [38], suggesting that the role of miR-34a is ambiguous in the regulation of taxane resistance. Some reports have shown that increased Bcl-2 expression is beneficial for breast cancer treatment [60]. On the other hand, miR-34a also has abundance of other targeted miRNAs besides Bcl-2, including SIRT-1, Cyclin D1, Cyclin E2, CDK4, CDK6, E2F3, MET and notch-1 [55], which add to the complexity of the role of miR-34a in human cancers. Bcl-2 antagonist killer 1, Bak1, is a pro-apoptotic member of Bcl-2 family. Bak1 is also a direct target of miR-125b, and restoring Bak1 expression in breast cancer cells could recover paclitaxel sensitivity, overcoming miR-125b-mediated paclitaxel resistance [61]. Bim is a member of the BH3-only family of pro-apoptotic proteins. In the ovarian cancer cells, miR-17–92 could induce paclitaxel resistance through targeting Bim [62]. Also, miR-101 overexpression in NSCLC cells promoted paclitaxel-induced apoptosis through down-regulating Enhancer of zeste homologue 2 (EZH2) expression, which has been shown to regulate apoptosis through epigenetically modulating Bim expression [63].

Despite being activated by different stimuli, both the intrinsic and the extrinsic apoptosis pathways result in the activation of casapase-3, the ultimate effector casapase, which can be regulated by specific miRNAs. In particular, by targeting caspase-3, let-7a directly regulated paclitaxel-induced apoptosis in HCC and human squamous carcinoma [64]. Accumulated evidence has demonstrated that increased activation of the phosphatidylinositol 3-kinase (PI3K)/Akt signalling pathway is responsible for resistance to taxanes. Once activated, Akt phosphorylates and inhibits several pro-apoptotic proteins such as Bim, Bad and caspase-9 [65]. Recent research showed that overexpression of miR-203 significantly sensitized the cytotoxicity of paclitaxel in the p53-mutated colon cancer cells by negatively regulating Akt2 expression [66]. Phosphatase and tensin homologue (PTEN), another important tumour suppressor gene, inhibits Akt by dephosphorylating phosphoinositide 3-phosphatase (PIP3) and acts as a negative regulator of PI3K/Akt signalling. Loss of PTEN activity leads to increased cell growth and overactivation of the PI3K/Akt pathway [65]. Overexpression of miR-22 enhanced the anti-cancer effect of paclitaxel in the p53-mutated cells through increasing cell apoptosis and reducing cell proliferation and survival, mediated by activation of PTEN signalling [67].

MiR-21 has been described as an oncogenic miRNA, and increased level of miR-21 expression has been observed in different cancer types, including breast cancer, prostate cancer, NSCLC, glioblastoma and hepatocellular carcinoma [68]. MiR-21 functions mainly through repression of two targets. One is programmed cell death 4 (PDCD4), a novel suppressor of tumourigenesis, tumour progression and invasion [69]. A recent study has demonstrated that PDCD4 negatively regulates YB-1 expression *via* its interaction with Twist1 and is involved in cancer cell growth and resistance to cisplatin and paclitaxel [70]. MiR-21 contributed to the resistance of prostate cancer cells to docetaxel by targeting PDCD4 [71]. The second is the tumour suppressor PTEN. MiR-21 modulated chemosensitivity by targeting PTEN in NSCLC [72] and breast cancer [73]. However, depletion of miR-21 neither resulted in change in downstream targets like PDCD4 and PTEN, nor affected the response to paclitaxel in prostate cancer cells [74]. These data suggested that the oncogenic properties of miR-21 could be cell- and tissue-dependent. In another report, miR-21 was shown to participate in enhancing sensitivity of human glioblastoma cells to paclitaxel by inhibiting STAT3 expression, independent of PTEN status [75].

Mitogen-activated protein kinase pathway comprises several key signalling components, which play a role in tumourigenesis and chemoresistance [76, 77]. Oncogene KRAS is a key molecule of EGFR/RAS/MAPK pathway, and miR-143 can restore chemosensitivity to docetaxel in prostate cancer cells by down-regulating KRAS [78]. On the other hand, overexpression of miR-148a induced paclitaxel resistance in prostate cancer cells by targeting MSK-1(Mitogen- and stress-activated kinase 1), a down-stream target of ERK or p38 MAPK [79].

### MiRNAs involved in regulation of drug transports

Multidrug resistance (MDR) is a phenomenon that resistance to one drug can result in cross-resistance to other structurally unrelated drugs. A key mechanism underlying multidrug resistance relates to the expression of the ATP-dependent transporter family known as the ATP-binding cassette (ABC) family. The human genome contains 48 genes that encode ABC transporters, which have been divided into seven subfamilies labelled A–G [80]. The ABC transporters involved in taxane resistance include P-gp (ABCB1), ABCC2, ABCC10 and ABCB11 [81].

P-glycoprotein (P-gp) is the best known and well-studied multidrug transporter, encoded by the MDR1 gene. Overexpression of P-gp confers cancer cell resistance to a broad spectrum of drugs, including colchicine, doxorubicin, etoposide, vinblastine and paclitaxel [82]. The level of MDR1 expression is correlated with resistance to paclitaxel in some cancers, such as lung cancer and breast cancer [83, 84]. MiR-451, miR-27, miR-326 and miR-331-5p have been shown to regulate the expression of the MDR1 gene [85–88]. In particular, miR-27a was found to be involved in the development of paclitaxel resistance, partly by targeting Homoeodomain-interacting protein kinase-2 (HIPK2), which can lead to suppression of MDR1 gene by inhibiting hypoxia inducible factor-1α (HIF-1α) [89]. Also, let-7 g was shown to affect the sensitivity of a paclitaxel-resistant ovarian cancer cell by targeting IGF-II miRNA-binding protein 1(IMP-1), which stabilized and protected MDR1 miRNA as a RNA binding protein [90].

### MiRNA involved in regulation of EMT and cancer stem cell

Epithelial–mesenchymal transition is an essential developmental process by which cells get increased migratory behaviour by the loss of epithelial characteristics and the acquisition of a mesenchymal phenotype. Emerging evidence suggests that EMT may be responsible for cancer cell progression, invasion, metastasis and possibly, treatment resistance [91]. Phenotypic and molecular changes consistent with EMT have been observed in paclitaxel-resistant epithelial ovarian carcinoma, breast cancer and docetaxel-resistant prostate cancer [92]. However, the mechanisms by which the EMT regulates resistance to chemotherapeutic agents are not very well elucidated. Recently, miRNAs have been found to be involved in the acquisition and maintenance of EMT-type cells, which may play important roles in drug resistance and metastasis [93]. The docetaxel-resistant lung adenocarcinoma cell lines (SPC-A1/DTX and H1299/DTX) previously established by our laboratorydisplayed chemoradioresistance and mesenchymal features with enhanced invasiveness and motility, and reintroduction of let-7c could reverse their chemoradioresistance and EMT phenotypes [94]. Low or absent miR-200c was found in paclitaxel-resistant breast cancer, and the loss of the miR-200c was critical for the acquisition of EMT characteristics mediated by Zinc-finger enhancer binding (ZEB) transcription factors (ZEB1 and ZEB2) [18], which could induce EMT phenotype by suppressing the expression of many epithelial genes including E-cadherin, and form a feedback loop with miR-200 family [95].

Recent evidence suggests that EMT cells have cancer stem cell-like features and cancer stem cells (CSCs) exhibit a mesenchymal-like phenotype, highlighting a link between EMT and the CSCs. Increased stem cell-like properties and invasion potential could be seen in the docetaxel-resistant prostate cancer cells, and the E-cadherin loss in these cells was partially owing to the decreased expression of miR-200c and miR-205 [96]. Up-regulation of miR-125b mediated by the overexpression of Snail could enhance the resistance to paclitaxel and increase the CSC population (CD24^−^CD44^+^), through repressing Bak1 [97]. Lin28, a marker of CSCs, might attenuate the sensitivity to paclitaxel treatment of breast cancer cells by affecting the let-7 processing [98], but the mechanisms are still poorly understood.

Apart from these mentioned above, miRNAs can also regulate taxane response through other mechanisms. The amplification of HGF receptor tyrosine kinase MET has been shown to mediate the resistance to EGFR-TKI in NSCLC. Recent research has found that miR-31-dependent regulation of MET was responsible for the resistance to paclitaxel of ovarian cancer [99]. A distinct miRNA profiling was shown in the paclitaxel-resistant ovarian cancer, and particularly down-regulation of miR-130a was associated with the translational activation of the macrophage colony-stimulating factor (M-CSF) gene, a known resistance factor for ovarian cancer [100], possibly owing to the role of matrix metalloproteinases (MMPs) in the CSF-1-mediated effect on tumour progression [101].

## Discussion

The expression signature of many miRNAs is tissue-, tumour- or even pathology-specific, and related to treatment response or clinical outcome. These features make miRNA appealing as a diagnostic and prognostic tool. MicroRNA profiling has been performed to explore its relationship with the taxane treatment response in NSCLC, ovarian cancer, breast cancer, and head and neck squamous cell carcinoma [100, 102–106]. For example, low expression of let-7a was significantly linked to better survival and response to the addition of paclitaxel to platinum-based chemotherapy of EOC patients [107]. In particular, tumour-derived miRNAs in circulation have raised great interest. Recent research showed that malignant effusion supernatant had a different profile of miRNAs compared with benign samples and the expression levels of cell-free miR-152 might discriminate docetaxel-sensitive samples from resistant ones [108]. Additionally, serum miR-21 levels were elevated in hormone-refractory prostate cancer patients, especially in those resistant to docetaxel-based chemotherapy [109]. Many efforts have been made in promoting miRNA-based therapies and there are already some encouraging results. However, a great number of challenges still remain to be overcome, including effective targeted delivery strategies, as well as off-target effects and long-term safety. So far, the miRNA-based treatments of reversing taxane resistance have not been reported.

## Conclusion

MicroRNAs have been demonstrated to regulate almost all the known processes and their dysregulation is involved in the mechanisms of carcinogenesis and ever more, drug resistance. In this review, we discuss recent studies which demonstrate that miRNAs could play a critical role in the regulation of taxane resistance (Table 1) and targeting miRNAs is a novel strategy for improving response to taxane.

**Table 1:** MiRNAs involved in pathways affecting taxanes sensitivity

microRNA	Target gene	Function	Drug	Tissue type	Mechanism	Refs.
miR-200c	TUBB3 (β III-tubulin)	Tumour suppressor	Paclitaxel	Ovarian cancer	Microtubule system	[19]
miR-100	TUBB2A (β 2A-tubulin), TUBB3 (β III-tubulin)	Tumour suppressor	Paclitaxel	Breast cancer	Microtubule system	[20]
miR-34c-5p	MAPT (Microtubule-associated protein tau)	Tumour suppressor	Paclitaxel	Gastric cancer	Microtubule system	[22]
miR-433	MAD2 (mitotic arrest deficiency protein 2)	Oncogene	Paclitaxel	Ovarian cancer	Cell cycle	[29]
let-7i	Cyclin D1 and D2 *etc*.	Tumour suppressor	Paclitaxel	Ovarian cancer	Cell cycle	[39]
miR-100	plk-1 (polo-like kinase 1)	Tumour suppressor	Docetaxel	NSCLC	Cell cycle	[36]
miR-200b	E2F3	Tumour suppressor	Docetaxel	NSCLC	Cell cycle	[41]
miR-135a	APC (Adenomatous polyposis coligene)	Oncogene	Paclitaxel	NSCLC	Cell cycle	[45]
miR-34a	Cyclin D1Bcl-2	Oncogene	Docetaxel	Breast cancer	Cell cycleapoptosis	[38]
	SIRT1 (Silent mating type information regulation 2homologue 1)	Tumour suppressor	Paclitaxel	Prostate cancer	Apoptosis	[59]
miR-34c	Bmf, c-myc	Oncogene	Paclitaxel	NSCLC	Apoptosis	[56]
miR-125b	Bak-1 (Bcl-2 antagonist killer 1)	Oncogene	Paclitaxel	Breast cancer	Apoptosis	[61]
miR-17–92	Bim	Oncogene	Paclitaxel	Ovarian cancer	Apoptosis	[62]
miR-101	EZH2 (Enhancer of zeste homologue 2)	Tumour suppressor	Paclitaxel	NSCLC	Apoptosis	[63]
Let-7a	Caspase-3	Oncogene	Paclitaxel	Squamous carcinoma, hepatocellular carcinoma	Apoptosis	[64]
miR-203	Akt-2	Tumour suppressor	Paclitaxel	Colon cancer	Apoptosis	[66]
miR-22	MTDH (Metadherin)	Tumour suppressor	Paclitaxel	Colon cancer	Apoptosis	[67]
miR-21	PDCD4 (Programmed cell death 4)	Oncogene	Docetaxel	Prostate cancer	Apoptosis	[71]
	PTEN (Phosphatase and tensin homologue)	Oncogene	Paclitaxel	Prostate cancer	Apoptosis	[74]
	STAT3	Oncogene	Paclitaxel	Glioblastoma multiforme	Apoptosis	[75]
miR-143	KRAS	Tumour suppressor	Docetaxel	Prostate cancer	Proliferation	[78]
miR-148a	MSK-1 (Mitogen- and stress-activated kinase 1)	Tumour suppressor	Paclitaxel	Prostate cancer	Apoptosis	[79]
miR-27a	HIPK2 (Homoeodomain-interacting protein kinase-2)	Tumour suppressor	Paclitaxel	Ovarian cancer	MDR	[89]
let-7g	IMP-1 (IGF-II miRNA-binding protein 1)	Tumour suppressor	Paclitaxel	Ovarian cancer	MDR	[90]
let-7c	Bcl-xL	Tumour suppressor	Docetaxel	NSCLC	EMT	[94]
miR-200c and miR-205	ZEB1 (Zinc-finger enhancer binding)	Tumour suppressor	Paclitaxel	Prostate cancer	EMT	[96]
miR-125b	Bak-1 ((Bcl-2 antagonist killer 1))	Oncogene	Paclitaxel		EMT, CSCs	[97]
miR-200c	ZEB1, ZEB2, TUBB3	Tumour suppressor	Paclitaxel	Endometrial, breast and ovarian cancer	EMT, microtubule system	[18]
miR-31	MET	Tumour suppressor	Paclitaxl	Ovarian cancer	Other	[99]
miR-130	MCSF (Macrophage colony-stimulating factor)	Tumour suppressor	Paclitaxel	Ovarian cancer	Other	[100]

## Expert commentary

Chemoresistance poses an obstacle to improving the survival rates of cancer cells. It is still difficult to find effective ways to overcome resistance before we can elucidate the complex and multilayered mechanisms. MicroRNAs have raised much interest owing to their unique and powerful regulatory capacity. For example, recent research studies have discovered a great number of miRNAs that can predict or regulate the response to taxanes of cancers. In addition, the research of miRNAs is rapidly advancing towards *in vivo* delivery for therapeutic purposes, ultimately building a new class of targeted molecular therapies. Over the past decade, strategies and techniques of miRNA-based therapy have developed greatly. Blocking oncogenic miRNAs can be achieved by the use of antisense oligonucleotides, miRNA sponges, miR-mask and small RNA inhibitors, whereas restoring the tumour suppressor miRNAs expression could be achieved by using synthetic miRNAs (miRNA mimics) or re-introducing genes coding for miRNAs by viral vectors [110]. Therapeutic silencing of miR-122 with a locked nucleic acid (LNA)-modified oligonucleotide in treatment of chronic hepatitis C virus has advanced into phase 2 clinical trials [111]. However, many questions remain unanswered, such as which miRNA is the best and most reliable to predict taxane response in cancer patients, and then, which target gene(s) is (are) the most critical to regulate the taxane response, and moreover, how to accurately deliver the miRNA to the tumour lesions *via* an effective and safe drug transport system.

## Five-year view

In the next 5 years, it is with great anticipation that miRNAs will have more profound clinical applications. MicroRNAs involved in specific networks, such as the apoptosis, cell cycle, or EMT-related signalling pathways, can influence the effects of anti-cancer treatments including taxanes, which highlights their potential values as biomarkers or modulators for anti-cancer drug response. We will see trials that explore the use of miRNAs or miRNA-based therapies for restoring chemosensitivity to some anti-cancer agents including taxanes. However, identification and verification of critical miRNA targets and lack of safe and specific delivery system still remain major difficulties to overcome. Based on a deeper understanding of the complex regulatory networks of miRNAs, the miRNA-based therapies hold great promise for overcoming drug resistance and developing more effective personalized anti-cancer treatment strategies.

## References

[1] Montero A, Fossella F, Hortobagyi G (2005). Docetaxel for treatment of solid tumours: a systematic review of clinical data. Lancet Oncol.

[2] Lujambio A, Lowe SW (2012). The microcosmos of cancer. Nature.

[3] Nana-Sinkam SP, Croce CM (2011). MicroRNAs as therapeutic targets in cancer. Transl Res.

[4] Ma J, Dong C, Ji C (2010). MicroRNA and drug resistance. Cancer Gene Ther.

[5] Gottesman MM (2002). Mechanisms of cancer drug resistance. Annu Rev Med.

[6] Downing KH, Nogales E (1999). Crystallographic structure of tubulin: implications for dynamics and drug binding. Cell Struct Funct.

[7] Abal M, Andreu JM, Barasoain I (2003). Taxanes: microtubule and centrosome targets, and cell cycle dependent mechanisms of action. Curr Cancer Drug Targets.

[8] Berrieman HK, Lind MJ, Cawkwell L (2004). Do beta-tubulin mutations have a role in resistance to chemotherapy?. Lancet Oncol.

[9] Burkhart CA, Kavallaris M, Band Horwitz S (2001). The role of beta-tubulin isotypes in resistance to antimitotic drugs. Biochim Biophys Acta.

[10] Orr GA, Verdier-Pinard P, Mcdaid H (2003). Mechanisms of Taxol resistance related to microtubules. Oncogene.

[11] Yin S, Bhattacharya R, Cabral F (2010). Human mutations that confer paclitaxel resistance. Mol Cancer Ther.

[12] Yusuf RZ, Duan Z, Lamendola DE (2003). Paclitaxel resistance: molecular mechanisms and pharmacologic manipulation. Curr Cancer Drug Targets.

[13] Mozzetti S, Ferlini C, Concolino P (2005). Class III beta-tubulin overexpression is a prominent mechanism of paclitaxel resistance in ovarian cancer patients. Clin Cancer Res.

[14] Pusztai L (2007). Markers predicting clinical benefit in breast cancer from microtubule-targeting agents. Ann Oncol.

[15] Seve P, Mackey J, Isaac S (2005). Class III beta-tubulin expression in tumor cells predicts response and outcome in patients with non-small cell lung cancer receiving paclitaxel. Mol Cancer Ther.

[16] Lee KM, Cao D, Itami A (2007). Class III beta-tubulin, a marker of resistance to paclitaxel, is overexpressed in pancreatic ductal adenocarcinoma and intraepithelial neoplasia. Histopathology.

[17] Kamath K, Wilson L, Cabral F (2005). BetaIII-tubulin induces paclitaxel resistance in association with reduced effects on microtubule dynamic instability. J Biol Chem.

[18] Cochrane DR, Spoelstra NS, Howe EN (2009). MicroRNA-200c mitigates invasiveness and restores sensitivity to microtubule-targeting chemotherapeutic agents. Mol Cancer Ther.

[19] Leskela S, Leandro-Garcia LJ, Mendiola M (2011). The miR-200 family controls beta-tubulin III expression and is associated with paclitaxel-based treatment response and progression-free survival in ovarian cancer patients. Endocr Relat Cancer.

[20] Lobert S, Jefferson B, Morris K (2011). Regulation of beta-tubulin isotypes by micro-RNA 100 in MCF7 breast cancer cells. Cytoskeleton (Hoboken).

[21] Wagner P, Wang B, Clark E (2005). Microtubule Associated Protein (MAP)-Tau: a novel mediator of paclitaxel sensitivity *in vitro* and *in vivo*. Cell Cycle.

[22] Wu H, Huang M, Lu M (2013). Regulation of microtubule-associated protein tau (MAPT) by miR-34c-5p determines the chemosensitivity of gastric cancer to paclitaxel. Cancer Chemother Pharmacol.

[23] Yvon AM, Wadsworth P, Jordan MA (1999). Taxol suppresses dynamics of individual microtubules in living human tumor cells. Mol Biol Cell.

[24] Lanzi C, Cassinelli G, Cuccuru G (2001). Cell cycle checkpoint efficiency and cellular response to paclitaxel in prostate cancer cells. Prostate.

[25] Fang G, Yu H, Kirschner MW (1999). Control of mitotic transitions by the anaphase-promoting complex. Philos Trans R Soc Lond B Biol Sci.

[26] Thornton BR, Toczyski DP (2003). Securin and B-cyclin/CDK are the only essential targets of the APC. Nat Cell Biol.

[27] Mcgrogan BT, Gilmartin B, Carney DN (2008). Taxanes, microtubules and chemoresistant breast cancer. Biochim Biophys Acta.

[28] Kienitz A, Vogel C, Morales I (2005). Partial downregulation of MAD1 causes spindle checkpoint inactivation and aneuploidy, but does not confer resistance towards taxol. Oncogene.

[29] Furlong F, Fitzpatrick P, O'toole S (2012). Low MAD2 expression levels associate with reduced progression-free survival in patients with high-grade serous epithelial ovarian cancer. J Pathol.

[30] Mcinnes C, Wyatt MD (2011). PLK1 as an oncology target: current status and future potential. Drug Discov Today.

[31] Uckun FM (2007). Chemosensitizing anti-cancer activity of LFM-A13, a leflunomide metabolite analog targeting polo-like kinases. Cell Cycle.

[32] Spankuch B, Kurunci-Csacsko E, Kaufmann M (2007). Rational combinations of siRNAs targeting Plk1 with breast cancer drugs. Oncogene.

[33] Peng DX, Luo M, Qiu LW (2012). Prognostic implications of microRNA-100 and its functional roles in human epithelial ovarian cancer. Oncol Rep.

[34] Shi W, Alajez NM, Bastianutto C (2010). Significance of Plk1 regulation by miR-100 in human nasopharyngeal cancer. Int J Cancer.

[35] Li BH, Zhou JS, Ye F (2011). Reduced miR-100 expression in cervical cancer and precursors and its carcinogenic effect through targeting PLK1 protein. Eur J Cancer.

[36] Feng B, Wang R, Chen LB (2012). MiR-100 resensitizes docetaxel-resistant human lung adenocarcinoma cells (SPC-A1) to docetaxel by targeting Plk1. Cancer Lett.

[37] Resnitzky D, Gossen M, Bujard H (1994). Acceleration of the G1/S phase transition by expression of cyclins D1 and E with an inducible system. Mol Cell Biol.

[38] Kastl L, Brown I, Schofield AC (2012). miRNA-34a is associated with docetaxel resistance in human breast cancer cells. Breast Cancer Rer.

[39] Liu N, Zhou C, Zhao J (2012). Reversal of paclitaxel resistance in epithelial ovarian carcinoma cells by a MUC1 aptamer-let-7i chimera. Cancer Invest.

[40] Ren B, Cam H, Takahashi Y (2002). E2F integrates cell cycle progression with DNA repair, replication, and G(2)/M checkpoints. Genes Dev.

[41] Feng B, Wang R, Song HZ (2012). MicroRNA-200b reverses chemoresistance of docetaxel-resistant human lung adenocarcinoma cells by targeting E2F3. Cancer.

[42] Giles RH, Van Es JH, Clevers H (2003). Caught up in a Wnt storm: Wnt signaling in cancer. Biochim Biophys Acta.

[43] Aoki K, Taketo MM (2007). Adenomatous polyposis coli (APC): a multi-functional tumor suppressor gene. J Cell Sci.

[44] Radulescu S, Ridgway RA, Appleton P (2010). Defining the role of APC in the mitotic spindle checkpoint *in vivo*: APC-deficient cells are resistant to Taxol. Oncogene.

[45] Holleman A, Chung I, Olsen RR (2011). miR-135a contributes to paclitaxel resistance in tumor cells both *in vitro* and *in vivo*. Oncogene.

[46] Torres K, Horwitz SB (1998). Mechanisms of taxol-induced cell death are concentration dependent. Cancer Res.

[47] Bacus SS, Gudkov AV, Lowe M (2001). Taxol-induced apoptosis depends on MAP kinase pathways (ERK and p38) and is independent of p53. Oncogene.

[48] Blagosklonny MV, Schulte TW, Nguyen P (1995). Taxol induction of p21WAF1 and p53 requires c-raf-1. Cancer Res.

[49] Blagosklonny MV, Giannakakou P, Eldeiry WS (1997). Raf-1/bcl-2 phosphorylation: a step from microtubule damage to cell death. Cancer Res.

[50] Wang TH, Wang HS, Ichijo H (1998). Microtubule-interfering agents activate c-Jun N-terminal kinase stress-activated protein kinase through both ras and apoptosis signal-regulating kinase pathways. J Biol Chem.

[51] Yamamoto K, Ichijo H, Korsmeyer SJ (1999). BCL-2 is phosphorylated and inactivated by an ASK1/Jun N-terminal protein kinase pathway normally activated at G(2)/M. Mol Cell Biol.

[52] Nimmanapalli R, Perkins CL, Orlando M (2001). Pretreatment with paclitaxel enhances Apo-2 ligand tumor necrosis factor-related apoptosis-inducing ligand-induced apoptosis of prostate cancer cells by inducing death receptors 4 and 5 protein levels. Cancer Res.

[53] Chen F, Zhu HH, Zhou LF (2010). Inhibition of c-FLIP expression by miR-512-3p contributes to taxol-induced apoptosis in hepatocellular carcinoma cells. Oncol Rep.

[54] Thorburn A (2004). Death receptor-induced cell killing. Cell Signal.

[55] Hermeking H (2010). The miR-34 family in cancer and apoptosis. Cell Death Differ.

[56] Catuogno S, Cerchia L, Romano G (2013). miR-34c may protect lung cancer cells from paclitaxel-induced apoptosis. Oncogene.

[57] Hotchkiss RS, Strasser A, Mcdunn JE (2009). Cell death. N Engl J Med.

[58] Ji Q, Hao XB, Meng Y (2008). Restoration of tumor suppressor miR-34 inhibits human p53-mutant gastric cancer tumorspheres. BMC Cancer.

[59] Kojima K, Fujita Y, Nozawa Y (2010). MiR-34a attenuates paclitaxel-resistance of hormone-refractory prostate cancer PC3 cells through direct and indirect mechanisms. Prostate.

[60] Hori M, Nogami T, Itabashi M (1997). Expression of Bcl-2 in human breast cancer: correlation between hormone receptor status, p53 protein accumulation and DNA strand breaks associated with apoptosis. Pathol Int.

[61] Zhou M, Liu Z, Zhao Y (2010). MicroRNA-125b confers the resistance of breast cancer cells to paclitaxel through suppression of pro-apoptotic Bcl-2 antagonist killer 1 (Bak1) expression. J Biol Chem.

[62] Shuang T, Shi C, Chang S (2013). Downregulation of miR-17–92 expression increase paclitaxel sensitivity in human ovarian carcinoma SKOV3-TR30 cells *via* BIM instead of PTEN. Int J Mol Sci.

[63] Zhang JG, Guo JF, Liu DL (2011). MicroRNA-101 exerts tumor-suppressive functions in non-small cell lung cancer through directly targeting enhancer of zeste homolog 2. J Thorac Oncol.

[64] Tsang WP, Kwok TT (2008). Let-7a microRNA suppresses therapeutics-induced cancer cell death by targeting caspase-3. Apoptosis.

[65] Mccubrey JA, Steelman LS, Abrams SL (2006). Roles of the RAF/MEK/ERK and PI3K/PTEN/AKT pathways in malignant transformation and drug resistance. Adv Enzyme Regul.

[66] Li J, Chen Y, Zhao J (2011). miR-203 reverses chemoresistance in p53-mutated colon cancer cells through downregulation of Akt2 expression. Cancer Lett.

[67] Li J, Zhang Y, Zhao J (2011). Overexpression of miR-22 reverses paclitaxel-induced chemoresistance through activation of PTEN signaling in p53-mutated colon cancer cells. Mol Cell Biochem.

[68] Krichevsky AM, Gabriely G (2009). miR-21: a small multi-faceted RNA. J Cell Mol Med.

[69] Pan X, Wang ZX, Wang R (2011). MicroRNA-21: a novel therapeutic target in human cancer. Cancer Biol Ther.

[70] Shiota M, Izumi H, Tanimoto A (2009). Programmed cell death protein 4 down-regulates Y-box binding protein-1 expression *via* a direct interaction with Twist1 to suppress cancer cell growth. Cancer Res.

[71] Shi GH, Ye DW, Yao XD (2010). Involvement of microRNA-21 in mediating chemo-resistance to docetaxel in androgen-independent prostate cancer PC3 cells. Acta Pharmacol Sin.

[72] Gao W, Lu X, Liu L (2012). MiRNA-21: a biomarker predictive for platinum-based adjuvant chemotherapy response in patients with non-small cell lung cancer. Cancer Biol Ther.

[73] Wang ZX, Lu BB, Wang H (2011). MicroRNA-21 modulates chemosensitivity of breast cancer cells to doxorubicin by targeting PTEN. Arch Med Res.

[74] Folini M, Gandellini P, Longoni N (2010). miR-21: an oncomir on strike in prostate cancer. Mol Cancer.

[75] Ren Y, Zhou X, Mei M (2010). MicroRNA-21 inhibitor sensitizes human glioblastoma cells U251 (PTEN-mutant) and LN229 (PTEN-wild type) to taxol. BMC Cancer.

[76] Santarpia L, Lippman SM, El-Naggar AK (2012). Targeting the MAPK-RAS-RAF signaling pathway in cancer therapy. Expert Opin Ther Targets.

[77] Haagenson KK, Wu GS (2010). The role of MAP kinases and MAP kinase phosphatase-1 in resistance to breast cancer treatment. Cancer Metastasis Rev.

[78] Xu B, Niu X, Zhang X (2011). miR-143 decreases prostate cancer cells proliferation and migration and enhances their sensitivity to docetaxel through suppression of KRAS. Mol Cell Biochem.

[79] Fujita Y, Kojima K, Ohhashi R (2010). MiR-148a attenuates paclitaxel resistance of hormone-refractory, drug-resistant prostate cancer PC3 cells by regulating MSK1 expression. J Biol Chem.

[80] Szakacs G, Paterson JK, Ludwig JA (2006). Targeting multidrug resistance in cancer. Nat Rev Drug Discov.

[81] Childs S, Yeh RL, Hui D (1998). Taxol resistance mediated by transfection of the liver-specific sister gene of P-glycoprotein. Cancer Res.

[82] Dean M, Fojo T, Bates S (2005). Tumour stem cells and drug resistance. Nat Rev Cancer.

[83] Maraz A, Furak J, Palfoldi R (2011). Roles of BCL-2 and MDR1 expression in the efficacy of paclitaxel-based lung cancer chemoradiation. Anticancer Res.

[84] Mechetner E, Kyshtoobayeva A, Zonis S (1998). Levels of multidrug resistance (MDR1) P-glycoprotein expression by human breast cancer correlate with *in vitro* resistance to taxol and doxorubicin. Clin Cancer Res.

[85] Kovalchuk O, Filkowski J, Meservy J (2008). Involvement of microRNA-451 in resistance of the MCF-7 breast cancer cells to chemotherapeutic drug doxorubicin. Mol Cancer Ther.

[86] Zhu H, Wu H, Liu X (2008). Role of MicroRNA miR-27a and miR-451 in the regulation of MDR1/P-glycoprotein expression in human cancer cells. Biochem Pharmacol.

[87] Liang ZX, Wu H, Xia J (2010). Involvement of miR-326 in chemotherapy resistance of breast cancer through modulating expression of multidrug resistance-associated protein 1. Biochem Pharmacol.

[88] Feng DD, Zhang H, Zhang P (2011). Down-regulated miR-331-5p and miR-27a are associated with chemotherapy resistance and relapse in leukaemia. J Cell Mol Med.

[89] Li Z, Hu S, Wang J (2010). MiR-27a modulates MDR1/P-glycoprotein expression by targeting HIPK2 in human ovarian cancer cells. Gynecol Oncol.

[90] Boyerinas B, Park SM, Murmann AE (2012). Let-7 modulates acquired resistance of ovarian cancer to Taxanes *via* IMP-1-mediated stabilization of multidrug resistance 1. Int J Cancer.

[91] Thiery JP, Sleeman JP (2006). Complex networks orchestrate epithelial-mesenchymal transitions. Nat Rev Mol Cell Biol.

[92] Mimeault M, Batra SK (2011). Frequent gene products and molecular pathways altered in prostate cancer- and metastasis-initiating cells and their progenies and novel promising multitargeted therapies. Mol Med.

[93] Singh A, Settleman J (2010). EMT, cancer stem cells and drug resistance: an emerging axis of evil in the war on cancer. Oncogene.

[94] Cui SY, Huang JY, Chen YT (2013). Acquisition of chemo- or radioresistance and epithelial to mesenchymal transition (EMT) Phenotypes in docetaxel-resistant lung adenocarcinoma cells was linked with downregulation of let-7c. Mol Cancer Res.

[95] Brabletz S, Brabletz T (2010). The ZEB/miR-200 feedback loop–a motor of cellular plasticity in development and cancer?. EMBO Rep.

[96] Puhr M, Hoefer J, Schafer G (2012). Epithelial-to-mesenchymal transition leads to docetaxel resistance in prostate cancer and is mediated by reduced expression of miR-200c and miR-205. Am J Pathol.

[97] Liu Z, Liu H, Desai S (2013). miR-125b functions as a key mediator for snail-induced stem cell propagation and chemoresistance. J Biol Chem.

[98] Lv K, Liu L, Wang L (2012). Lin28 mediates paclitaxel resistance by modulating p21, Rb and let-7a miRNA in breast cancer cells. PLoS ONE.

[99] Mitamura T, Watari H, Wang L (2013). Downregulation of miRNA-31 induces taxane resistance in ovarian cancer cells through increase of receptor tyrosine kinase MET. Oncogenesis.

[100] Sorrentino A, Liu CG, Addario A (2008). Role of microRNAs in drug-resistant ovarian cancer cells. Gynecol Oncol.

[101] Paulus P, Stanley ER, Schafer R (2006). Colony-stimulating factor-1 antibody reverses chemoresistance in human MCF-7 breast cancer xenografts. Cancer Res.

[102] Dai Y, Xie CH, Neis JP (2011). MicroRNA expression profiles of head and neck squamous cell carcinoma with docetaxel-induced multidrug resistance. Head Neck.

[103] Rui W, Bing F, Hai-Zhu S (2010). Identification of microRNA profiles in docetaxel-resistant human non-small cell lung carcinoma cells (SPC-A1). J Cell Mol Med.

[104] Boren T, Xiong Y, Hakam A (2009). MicroRNAs and their target messenger RNAs associated with ovarian cancer response to chemotherapy. Gynecol Oncol.

[105] Salter KH, Acharya CR, Walters KS (2008). An integrated approach to the prediction of chemotherapeutic response in patients with breast cancer. PLoS ONE.

[106] Li X, Lu Y, Chen Y (2013). MicroRNA profile of paclitaxel-resistant serous ovarian carcinoma based on formalin-fixed paraffin-embedded samples. BMC Cancer.

[107] Lu L, Schwartz P, Scarampi L (2011). MicroRNA let-7a: a potential marker for selection of paclitaxel in ovarian cancer management. Gynecol Oncol.

[108] Xie L, Chen X, Wang L (2010). Cell-free miRNAs may indicate diagnosis and docetaxel sensitivity of tumor cells in malignant effusions. BMC Cancer.

[109] Zhang HL, Yang LF, Zhu Y (2011). Serum miRNA-21: elevated levels in patients with metastatic hormone-refractory prostate cancer and potential predictive factor for the efficacy of docetaxel-based chemotherapy. Prostate.

[110] Garzon R, Marcucci G, Croce CM (2010). Targeting microRNAs in cancer: rationale, strategies and challenges. Nat Rev Drug Discov.

[111] Lanford RE, Hildebrandt-Eriksen ES, Petri A (2010). Therapeutic silencing of microRNA-122 in primates with chronic hepatitis C virus infection. Science.

